# Plasma Adiponectin, Clinical Factors, and Patient Outcomes during the Acute Respiratory Distress Syndrome

**DOI:** 10.1371/journal.pone.0108561

**Published:** 2014-09-26

**Authors:** Allan J. Walkey, Serkalem Demissie, Dilip Shah, Freddy Romero, Leah Puklin, Ross S. Summer

**Affiliations:** 1 The Pulmonary Center, Boston University School of Medicine, Division of Pulmonary, Allergy and Critical Care Medicine, Boston Medical Center, Boston, Massachusetts, United States of America; 2 Department of Biostatistics, Boston University School of Public Health, Boston, Massachusetts, United States of America; 3 Center for Translational Medicine and Korman Lung Center, Thomas Jefferson University, Philadelphia, Pennsylvania, United States of America; Duke University Medical Center, United States of America

## Abstract

**Objective:**

Adiponectin (APN) is an anti-inflammatory hormone derived from adipose tissue that attenuates acute lung injury in rodents. In this study, we investigated the association between circulating APN and outcomes among patients with acute respiratory distress syndrome (ARDS).

**Methods:**

We performed a retrospective cohort study using data and plasma samples from participants in the multicenter ARDS Network Fluid and Catheter Treatment Trial.

**Results:**

Plasma APN concentrations were measured in 816 (81.6%) trial participants at baseline and in 568 (56.8%) subjects at both baseline and day 7 after enrollment. Clinical factors associated with baseline APN levels in multivariable-adjusted models included sex, body mass index, past medical history of cirrhosis, and central venous pressure (model R^2^ = 9.7%). We did not observe an association between baseline APN and either severity of illness (APACHE III) or extent of lung injury (Lung Injury Score). Among patients who received right heart catheterization (n = 384), baseline APN was inversely related to mean pulmonary artery pressure (β = −0.015, R^2^ 1.5%, p = 0.02); however, this association did not persist in multivariable models (β = −0.009, R^2^ 0.5%, p = 0.20). Neither baseline APN levels [HR per quartile1.04 (95% CI 0.91–1.18), p = 0.61], nor change in APN level from baseline to day 7 [HR 1.04 (95% CI 0.89–1.23), p = 0.62)] were associated with 60 day mortality in Cox proportional hazards regression models. However, subgroup analysis identified an association between APN and mortality among patients who developed ARDS from extra-pulmonary etiologies [HR per quartile 1.31 (95% CI 1.08–1.57)]. APN levels did not correlate with mortality among patients developing ARDS in association with direct pulmonary injury [HR 0.96 (95% CI 0.83–1.13)], p_interaction_ = 0.016.

**Conclusions:**

Plasma APN levels did not correlate with disease severity or mortality in a large cohort of patients with ARDS. However, higher APN levels were associated with increased mortality among patients developing ARDS from extra-pulmonary etiologies.

## Introduction

Adiponectin (APN) is a highly abundant circulating hormone with pleotrophic effects upon diverse pulmonary cell types [1]–[4] including alveolar macrophages, epithelium, and vascular endothelium [3], [5], [6]. Though principally derived from adipocytes, circulating APN levels are paradoxically increased in lean individuals and decreased in subjects with higher body mass indexes [7]. In the lung, APN has been shown to have important anti-inflammatory and vascular protective actions, prompting pre-clinical studies examining the role of APN in the pathogenesis of pulmonary disorders including acute respiratory distress syndrome (ARDS) [2]–[4], [8]

ARDS is an acute, life-threatening lung condition involving immune and endothelial cell activation that develops in association with direct (e.g. pneumonia) and indirect pulmonary (e.g., urosepsis) challenges [9]. Although the role of circulating APN in human ARDS has not yet been investigated, animal studies suggest a causal link between hypoadiponectemia and ARDS pathogenesis. For example, APN-deficient mice have a markedly increased susceptibility to developing acute lung injury (the murine equivalent of ARDS) [2], [4], [6], [8] and this susceptibility is attenuated following APN replacement [3]. Further, mice deficient in APN are prone to developing pulmonary vascular abnormalities including elevated pulmonary artery pressures [5], a hemodynamic alteration associated with increased mortality among patients with ARDS [10]. Together, these pre-clinical findings prompted our current investigation into the relationship of circulating APN to outcomes in ARDS.

## Materials and Methods

### Data Source

We used National Heart, Lung and Blood Institute (NHLBI) ARDS Network Fluid and Catheter Treatment Trial (FACTT) Trial [11], [12] research materials obtained from the NHLBI Biologic Specimen and Data Repository Information Coordinating Center (BioLINCC). The ARDS Network is comprised of 42 hospitals across the United States; details regarding protocols of ARDS Network studies can be found at the ARDSNet.org website. The FACTT trial was a 2×2 factorial design, which randomized patients with ARDS to receive either a right heart catheter or a central venous catheter [12] and either conservative or liberal fluid management [11], [13]. Patients receiving conservative fluid management had overall fewer days of mechanical ventilation but otherwise outcomes did not differ significantly between groups. For our study, all analyses and procedures were approved by the Boston University School of Medicine, the Thomas Jefferson University School of Medicine Institutional Review Boards and NHLBI BioLINCC.

### Covariates and outcomes

Baseline covariates were measured at FACTT enrollment, which was within 48 hours of the development of ARDS. Data obtained from trial participants included demographics (age, race, sex), anthropometrics (body mass index, height, weight), comorbid conditions (HIV/Acquired Immunodeficiency Syndrome, arthritis, chronic pulmonary disease, cirrhosis, dementia, diabetes mellitus, dialysis dependence, alcohol abuse, congestive heart failure, hepatic encephalopathy, hypertension, immunosuppression, leukemia, lymphoma, myocardial infarction, stroke, metastatic solid tumor, peptic ulcer, peripheral vascular disease), vital signs (blood pressure, heart rate, central venous pressure, fluid intake and output, PaO_2_/FiO_2_, vasopressor requirement), laboratory values (albumin, bicarbonate, blood urea nitrogen, chloride, creatinine, glucose, hemoglobin, platelets, potassium, sodium, protein and white blood cell count), Murray lung injury score (a composite radiographic appearance, oxygenation, PEEP level and lung compliance) [14], and baseline Acute Physiology and Chronic Health Evaluation (APACHE) III score [15]. Among patients randomized to receive a right heart catheterization, recordings of pulmonary artery pressures, pulmonary artery occlusion pressure, cardiac index and transpulmonary pressure gradient (mean pulmonary pressure minus pulmonary artery occlusion pressure) were also utilized. Patients were followed for up to 60 days for outcome of ‘death prior to discharge home with unassisted breathing’.

### Adiponectin measurements

Plasma APN concentration was measured by enzyme-linked immunosorbent assay using a commercially available kit (R&D Systems, Minneapolis, MN) according to company protocol. All plasma samples were diluted 1∶200 in serum diluting buffer prior to assessing APN concentrations.

### Statistical Analyses

The distribution of clinical variables among APN quartiles was compared using Kruskal-Wallis testing and Mantel-Haenzel tests, as appropriate. In order to determine clinical factors associated with APN, variables that were nominally associated with APN levels in univariate analyses (p<0.10) were entered into multivariable linear regression with backward selection (p<0.10). Given *a priori* associations of age with APN levels and clinical outcomes, age was forced into adjusted models that did not include APACHE scores (which are calculated using age). We excluded variables with >15% missing data from inclusion in multivariable models. APN and change in APN were log-transformed and used as dependent variables in multivariable linear regression analyses. We specifically explored the associations between APN and pulmonary hemodynamics, as well as APN and lung injury scores in multivariable models after adjusting for other clinical factors associated with APN. Separate analyses were performed for clinical factors at baseline and for the change in clinical variables from baseline to day 7. We explored the association of APN with 60-day mortality in Cox proportional hazards models using quartiles of APN levels and clinical variables associated with APN as independent variables. We examined the association of APN with 60-day mortality and quartiles of change in APN (among patients surviving to day 7) in a similar manner. Secondary models that included APN and other variables as time-varying covariates in Cox proportional hazards models were also evaluated. Because of the role of APN in attenuating endothelial activation in animal models of acute lung injury [3], we performed an exploratory subgroup analysis investigating the association between APN and mortality stratifying patients by either “direct” pulmonary injury (eg., pneumonia, aspiration) or “indirect” injury from extra-pulmonary etiologies (eg., urosepsis, trauma, transfusion) [16]. We used Kaplan-Meier survival plots with log-rank tests to visually demonstrate the association between APN and mortality and report 60day mortality rates generated from the Kaplan-Meier survival function. Finally, we examined Spearman rank correlations between APN and log-transformed ventilator-free days, a composite outcome of duration of mechanical ventilation and mortality [17]. In order to examine whether multiple hypothesis testing might better explain significant (at alpha = 0.05) associations between APN and clinical variables, univariate associations between clinical factors and APN levels were examined in a sensitivity analysis using a Bonferonni-adjusted threshold of p = 0.001 (derived from alpha 0.05/50 tests).

Given that a prior study of critically ill patients with respiratory failure demonstrated an odds ratio for 28-day mortality of 1.59 associated for each 5 ug/ml increase in total APN [18], we estimated a power of 82% with alpha = 0.05 to detect similar association in the current study. All analyses were performed using SAS 9.1 (Cary, NC, USA), with alpha of 0.05.

## Results

### Patient characteristics

Of 1000 FACTT trial participants, plasma was available from 827 subjects at baseline, of whom 816 (81.6%) had detectable APN levels, and from 568 (56.8%) subjects at both baseline and day 7 after enrollment. Trial participants were on average 49.8±16 years old, 53% were male, and 64% were white, with 60-day mortality of 26.8% (219/816). Compared to patients from whom plasma was available, the 184 subjects that were not studied had similar distributions of age (p = 0.92), sex (p = 1.0), race (p = 0.39), etiology of ARDS (p = 0.24), and APACHE III score (p = 0.10). Among the 248 patients who provided baseline but not day 7 plasma samples, 149 (60%) remained hospitalized at day 7, 24 (10%) were discharged home prior to day 7, and 75 (30%) died prior to day 7.

### Adiponectin and Clinical Factors

Analysis of plasma APN levels showed that median APN levels increased over the course of hospitalization: baseline [7245 pg/ml (IQR 3610–13288)], day 7 [9451 pg/ml (IQR 5038–15576)] and change from baseline to day 7 [1346 pg/ml (IQR −2194–5879), p<0.001]. Demographic and clinical characteristics grouped according to baseline APN quartiles are shown in **Table S1 in File S1**. Factors associated with baseline APN levels after inclusion in multivariable-adjusted models included sex, body mass index, past medical history of cirrhosis, and central venous pressure (**Table 1**, N = 666, model R^2^ = 9.7%). Baseline APN levels were not associated with APACHE III critical illness severity scores, Murray lung injury scores, or measures of oxygenation (PaO_2_/FiO_2_ ratio). Factors associated with change in APN level from baseline to day 7 are shown in **Table S2 in File S1**. In multivariable analysis, only a history of cirrhosis (β = −0.28, R^2^ = 0.84%, p<0.04) was associated with change in APN from baseline to day 7.

**Table 1:** Multivariable-adjusted model of factors associated with baseline adiponectin levels.

Variable	Beta estimate	Partial R^2^	p-value
Age (per year)	0.0045	0.005	0.069
Male sex	−0.3213	0.026	<0.0001
Body mass index	−0.022	0.027	<0.0001
AIDS	0.2626	0.005	0.083
Cirrhosis	0.6903	0.017	0.001
Central venous pressure	−0.0207	0.009	0.015
Positive end expiratory pressure	−0.0191	0.006	0.054

Model N = 666, Model R^2^ = 0.097.

**Table 2** demonstrates the association between baseline APN levels and pulmonary hemodynamic variables among 384 patients who were randomized to receive a pulmonary artery catheter. Baseline APN levels were inversely associated with mean pulmonary arterial pressure (β = −0.015, R^2^ = 1.5%, p = 0.02) but not with other measures of pulmonary vascular hemodynamics such as transpulmonary pressure, pulmonary artery occlusion pressure or cardiac index. After adjusting for age, race, sex, body mass index, and relevant clinical factors (ie., PEEP, cirrhosis, HIV) association between mean pulmonary artery pressure and baseline APN was attenuated (β = −0.009, R^2^ = 0.5%, p = 0.20).

**Table 2:** Pulmonary artery catheter hemodynamics and adiponectin levels.

	Baseline Adiponectin Quartile				
Pulmonary artery catheter value	Quartile 1 (95–3364 pg/ml) N = 96	Quartile 2 (3380–7164) N = 96	Quartile 3 (7202–13954) N = 96	Quartile 4 (13974–60336) N = 96	p
Pulmonary artery systolic pressure (mmHg)	42 (37–51)	40 (34–48)	39 (33–46)	41 (33–49)	0.038
Pulmonary artery diastolic pressure (mmHg)	23 (20–28)	21 (16–26)	22 (17–26)	21 (17–25)	0.013
Mean pulmonary artery pressure (mmHg)	30 (27–35)	27 (23–33)	27 (23–32)	28 (22–33)	0.01*
Pulmonary artery occlusion pressure (mmHg)	16 (12–20)	15 (10–18)	15 (12–18)	15 (11–18)	0.23
Trans-pulmonary pressure gradient (mm Hg)	14.3 (11.3–18)	13.5 (8.8–17)	12.7 (9.3–16.8)	12.7 (8.7–15.7)	0.12
Cardiac Index (l/min/m^2^)	4.1 (3.4–4.8)	3.8 (3.2–4.4)	3.7 (3.0–4.8)	4.1 (3.1–5.1)	0.23

Values are medians (Inter-Quartile Range).

Trans-pulmonary pressure gradient: Mean pulmonary artery pressure-Pulmonary artery occlusion pressure.

*p<0.1 and selected for inclusion in multivariable models.

### Adiponectin and Outcomes

**Figure 1:**
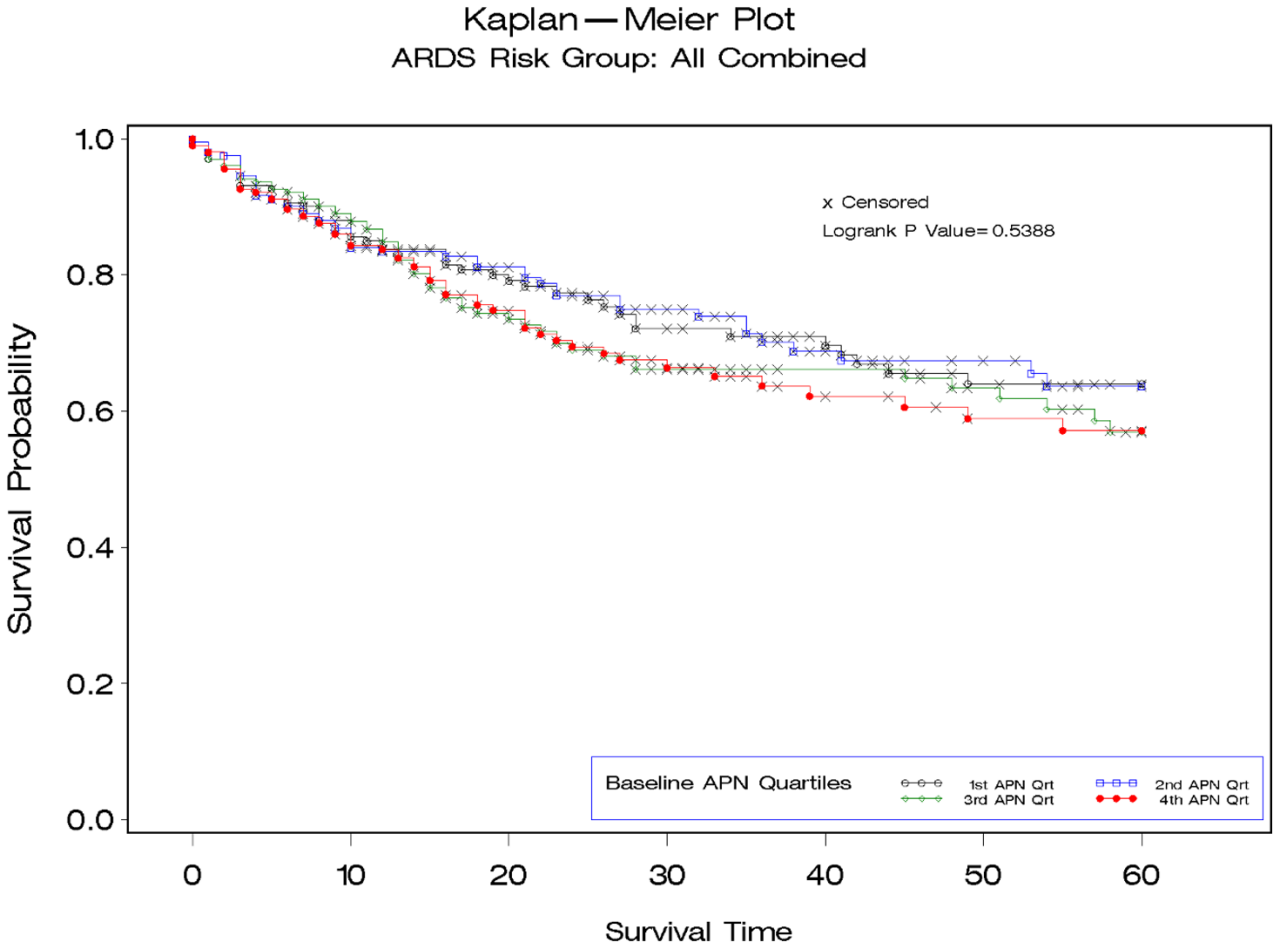
Kaplan-Meier survival plot demonstrating 60-day mortality for patients with acute respiratory distress syndrome stratified by quartile of baseline adiponectin (APN) level for full cohort (N = 816). APN Quartile 1: 95–3589 pg/ml; Quartile 2: 3630–7241 pg/ml; Quartile 3: 7248–13212; Quartile 4: 7248–13212.

When analyzing all subjects, neither baseline APN levels [APN quartile 4: 43% Kaplan-Meier mortality estimate vs. quartile 1: 36% mortality (**Figure 1**); HR per APN quartile1.04 (95% CI 0.91–1.18), p = 0.61;], change in APN from baseline to day 7 [HR 1.04 (0.89–1.23), p = 0.62)] nor analysis of APN as a time-varying covariate [HR for ln APN 1.13 (0.99–1.30) p = 0.07] were significantly associated with 60 day mortality. Further, we did not identify an association between baseline APN and ventilator-free days (R^2^ = 0.14%, p = 0.29).

**Figure 2:**
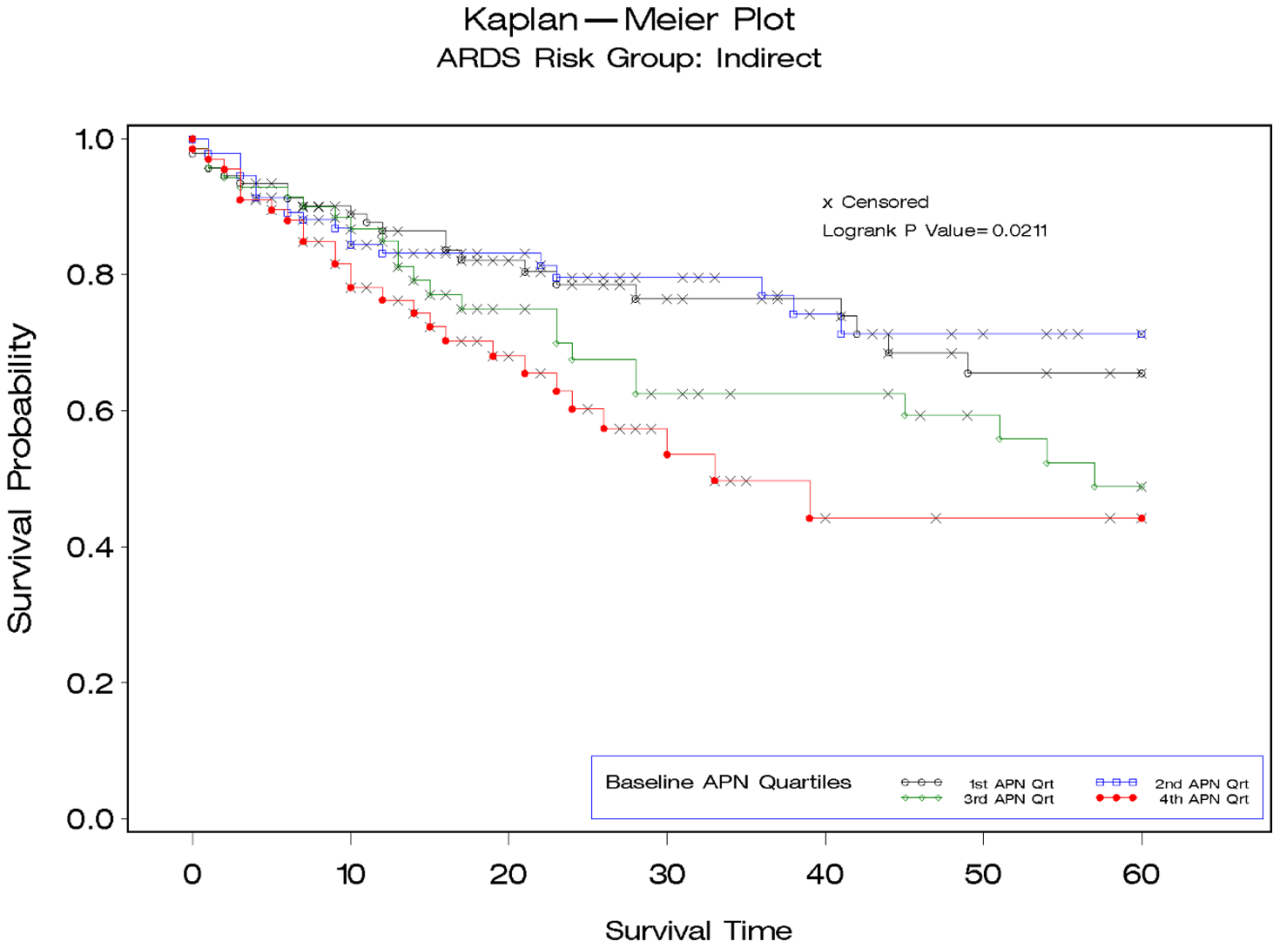
Kaplan-Meier survival plots demonstrating 60-day mortality for patients with acute respiratory distress syndrome stratified by quartile of baseline adiponectin (APN) level for patients with indirect lung injury (N = 322). A significant interaction was demonstrated (p = 0.016) for the association between baseline APN levels and mortality based on whether the mechanism of acute respiratory distress syndrome was presumed to be from “direct” pulmonary injury (eg., pneumonia, aspiration) or “indirect” injury from extra-pulmonary etiologies (eg., urosepsis, trauma, transfusion).

**Figure 3:**
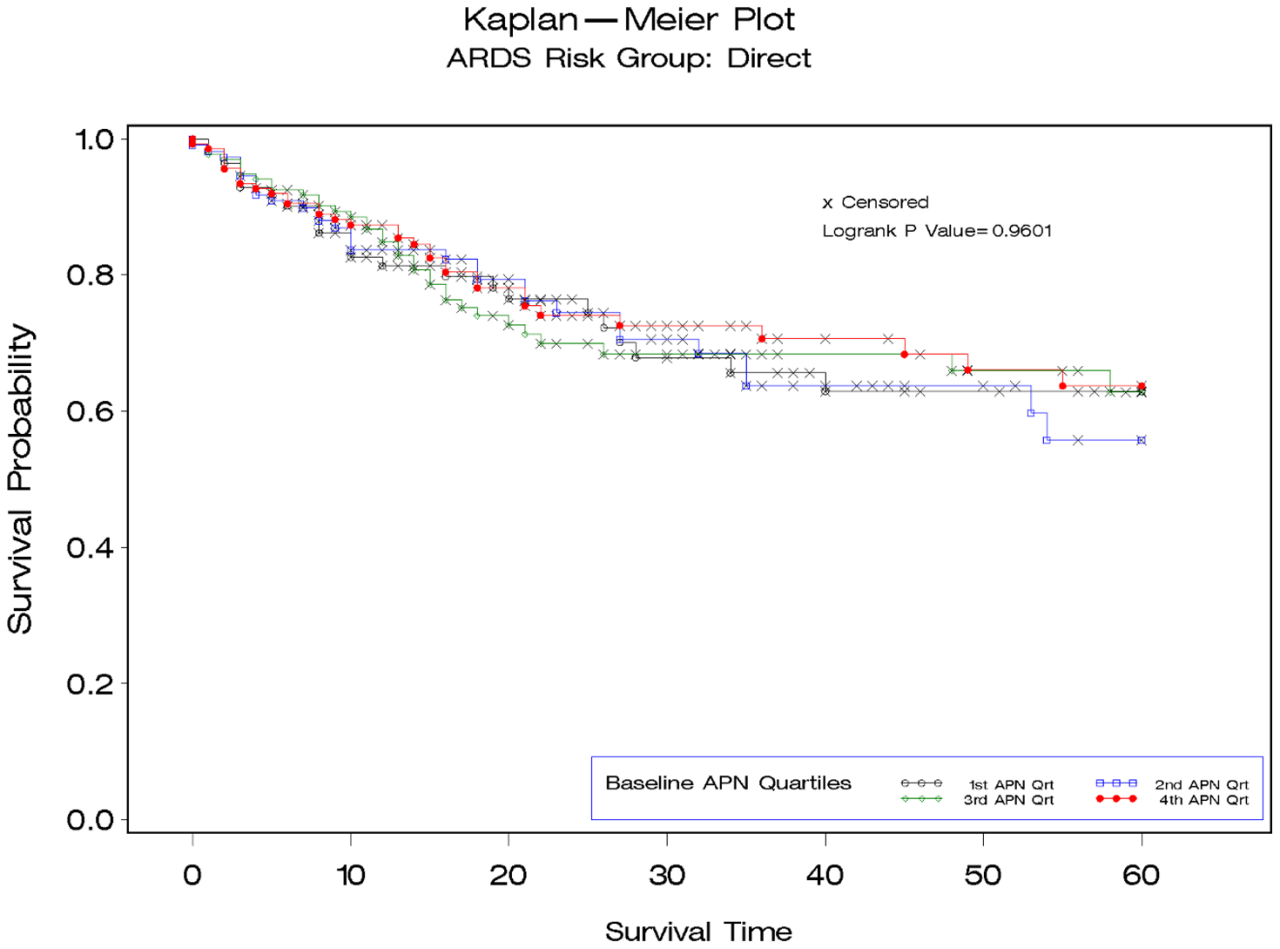
Kaplan-Meier survival plots demonstrating 60-day mortality for patients with acute respiratory distress syndrome stratified by quartile of baseline adiponectin (APN) level for patients with direct lung injury (N = 494).

To further explore the relationship between APN and mortality we stratified patients based on whether the etiology of ARDS came from direct or indirect lung injury. Patients with indirect lung injury [median 5909 pg/ml (IQR 3332–12127)] had significantly lower baseline APN levels than those with direct lung injury [7784 pg/ml (IQR 4122–14274)] p = 0.004. We observed a significant association between baseline APN levels and mortality among patients with indirect etiologies for ARDS [N = 322, APN quartile 4: 56% Kaplan-Meier mortality vs. quartile 1:34% (**Figure 2**); HR per APN quartile 1.31 (95% CI 1.08–1.57)]. In contrast, we did not identify an association between APN and mortality among patients developing ARDS in association with direct pulmonary injury [N = 494, APN quartile 4:36% Kaplan-Meier mortality vs. quartile 1: 37% (**Figure 3**); HR per APN quartile 0.96 (95% CI 0.83–1.13)], p interaction = 0.016. Among patients with indirect lung injuries, the association between baseline APN and mortality persisted after adjustment for age, race, sex, and body mass index [HR per APN quartile 1.29 (95% CI 1.05–1.58)], but this was attenuated after further adjusting for APACHE III score, central venous pressure and positive end expiratory pressure [HR per APN quartile 1.18(95% CI 0.94–1.49)]. No significant interaction was seen for the association between change in APN levels from baseline to day 7, mortality and mechanism of lung injury (p interaction = 0.63).

## Discussion

We investigated the clinical correlates and outcomes associated with plasma APN levels in more than 800 patients with well-classified ARDS. In addition to confirming previously-described associations between APN with sex and body mass index, we identified a novel correlation between APN levels and central venous pressures. We did not find a relationship between plasma APN levels and severity of illness or lung injury as measured by either the APACHE III score or the Murray Lung Injury score. Moreover, associations between APN and mean pulmonary arterial pressure did not persist after adjusting for demographic and clinical variables. Consistent with prior studies of critically ill patients [18], [19], we found that plasma APN levels were higher at late time points in ARDS when compared to the onset of disease. Surprisingly, neither baseline nor dynamic changes in APN levels were associated with mortality among all patients with ARDS in the present study. However, subgroup analysis showed an association between baseline APN levels (but not changes in APN) and mortality in patients who developed ARDS with extra-pulmonary etiologies.

Animal studies have demonstrated that hypoadiponectinemia predisposes to the onset of acute lung injury and increases the severity of disease [2]–[4], [8]. However, we are unaware of prior studies examining circulating APN levels, clinical factors, and outcomes in human ARDS. Ahasic et al recently showed that an APN gene polymorphism rs2082940 was associated with poor outcomes in ARDS, but circulating APN levels were not measured in this study [20]. In a single center cohort of 175 mechanically ventilated patients of diverse diagnoses (21% with ARDS), we previously showed that higher APN levels at study enrollment were associated with increased mortality [18]. Similarly, in a population of 170 critically ill patients Koch et al. reported that higher plasma APN levels measured early in the course disease were predictive of increased mortality [21]. In our current investigation we did not identify an association between plasma APN and mortality among all patients with ARDS, although we observed a positive correlation among patients with extra-pulmonary etiologies for ARDS. Our subgroup analysis may explain why previous studies have reported a positive association between APN and mortality among critically ill subjects, as extra-pulmonary conditions were more prevalent in the general intensive care unit populations in prior studies [18], [21].

The clinical observations that *higher* APN levels are associated with poor outcomes appear to conflict with experimental findings in rodents, which have consistently shown a relationship between *low* adiponectin states (ie, knockout mice) and poor outcomes in acute lung injury [2], [4], [6], [8]. However, in pre-clinical rodent studies (e.g. knock-out mice) changes in APN levels occur prior to the onset of disease, whereas in human studies APN levels are examined after disease onset. Thus, we postulate that higher APN levels in patients early after onset of an acute illness may represent a dysregulated immune response. We propose that future studies exploring this hypothesis focus on patients with extrapulmonary etiologies of ARDS, in whom APN-endothelial [3], [16] interactions may be of greater importance.

Our study confirmed prior associations of plasma APN levels with female sex, low body mass index, and cirrhosis [18], [21]. Additionally, we identified a novel association between plasma APN levels and central venous pressure. Although this observation was unanticipated, results are consistent with findings in other studies showing that APN acts on systemic blood vessels to increase nitric oxide production and decrease vascular tone [22]. We hypothesize that increased nitric oxide production might partly explain why lower central venous pressures were observed in patients with higher APN levels in our study.

This study has a number of strengths and several limitations. The most obvious strengths are that analyses were performed on a large number of samples and that detailed clinical characteristics were available for all subjects enrolled in our study. While a robust database provided us with sufficient power to detect moderate associations between APN and clinical factors, we recognize that more subtle associations might have been missed by our analyses. We also acknowledge that when testing multiple clinical factors to identify associations there is a greater risk of type I errors. However, our results would not have significantly changed with use of p-values corrected for multiple hypothesis tests. In addition, our analysis of the association between patient outcomes and changes in APN levels over time may have been affected by selection bias; patients without APN levels available on day 7 were more likely to have died prior to day 7 than patients available for sample collection. Measurement of daily APN may better demonstrate associations between temporal changes in APN and clinical outcomes in future studies. One other limitation of our study is that only total APN levels were measured in our samples. Since APN is known to circulate in at least three functionally distinct isoforms [23], [24] it is plausible that changes in individual APN isoforms may have more strongly correlated with outcomes in our study. In addition, analyses among subgroups of ARDS etiology were exploratory and should be considered hypothesis-generating. Lastly, as with all observational studies, causal inference for associations between clinical factors and APN levels is limited.

In conclusion, our study fills multiple knowledge gaps regarding the relationship between APN, clinical factors and clinical outcomes in ARDS. Although we did not observe significant associations between plasma APN and mortality in the entire cohort of patients, we identified novel clinical correlates of APN. Our analysis demonstrates an interaction between the mechanism of lung injury and the association of APN with mortality, a finding that is supported by experimental evidence and argues for further clinical investigation examining the role of APN in patients with indirect causes of ARDS.

## Supporting Information

File S1 **Legend. Table S1.** Baseline clinical and demographic characteristics of patients with ARDS according to baseline adiponectin quartile. **Table S2.** Characteristics Associated with Change in Adiponectin from Baseline (day 0) to Day 7.
(DOCX)
