# Supplementary material for: Plasma Adiponectin, Clinical Factors, and Patient Outcomes during the Acute Respiratory Distress Syndrome
Source: PLoS One. 2014 Sep 26;9(9):e108561. doi: 10.1371/journal.pone.0108561 (PMC4178176; doi:10.1371/journal.pone.0108561)
Supplement: File S1 — Legend. Table S1. Baseline clinical and demographic characteristics of patients with ARDS according to baseline adiponectin quartile. Table S2. Characteristics Associated with Change in Adiponectin from Baseline (day 0) to Day 7. (DOCX) [file pone.0108561.s001.docx]

**Supporting Information File S1**

**Plasma Adiponectin, Clinical Factors, and Patient Outcomes during the Acute Respiratory Distress Syndrome**

Allan J. Walkey, MD, MSc, Serkalem Demissie, PhD, Dilip Shah PhD, Freddy Romero PhD, Leah Puklin, and Ross S. Summer, MD

| **Table S1. Baseline clinical and demographic characteristics of patients with ARDS according to baseline adiponectin quartile.** | | | | | |
| --- | --- | --- | --- | --- | --- |
|  | **Baseline Adiponectin Quartile** | | | |  |
| **Characteristic**  Median (IQR) or  N (%) | **Quartile 1**  **( 95-3589 pg/ml)**  **N=204** | **Quartile 2**  **( 3630-7241)**  **N=204** | **Quartile 3**  **( 7248-13212)**  **N=204** | **Quartile 4**  **( 13364-60336)**  **N=204** | **p-value** |
| **Demographics/Anthropometrics** |  |  |  |  |  |
| Age (years)  N=816 | 47  (37-59) | 47  (35.5-59) | 50  (40-62) | 51.5  (40-63) | 0.028* |
| Race/Ethnicity |  |  |  |  | 0.06* |
| White | 123 (60) | 133 (65) | 140 (69) | 135 (66) |  |
| Black | 47 (23) | 40 (20) | 40 (20) | 46 (23) |  |
| Other | 34 (17) | 31 (15) | 24 (12) | 23 (11) |  |
| Sex (% male) | 121 (59) | 117 (57) | 104 (51) | 94 (46) | 0.003* |
| Body Mass Index (kg/cm^2^)  N=748 | 30.1  (25.2-35.5) | 27.8  (23.7-33.1) | 26.8  (23.2-31.0) | 24.5  (21.3-29.4) | <0.001* |
| Predicted Body weight (kg)  N=815 | 66.1  (55.0-73) | 66.1  (55.0-75.3) | 63.8  (54.7-71.0) | 61.5  (53.6-70.7) | 0.003 |
| Primary ARDS risk factor |  |  |  |  | 0.37 |
| Pneumonia | 91 (45) | 74 (36) | 100 (49) | 110 (54) |  |
| Sepsis | 58 (28) | 47 (23) | 47(23) | 48 (24) |  |
| Trauma | 21 (10) | 29 (14) | 6 (3) | 6 (3) |  |
| Aspiration | 21 (10) | 37 (18) | 34 (17) | 27 (13) |  |
| Transfusion | 2 (1) | 2 (1) | 4 (2) | 1 (0.5) |  |
| Other | 11 (5) | 15 (7) | 13 (6) | 12 (6) |  |
| **Comorbid Conditions** |  |  |  |  |  |
| AIDS | 7 (4) | 13(6) | 18(9) | 20(10) | 0.005* |
| Arthritis | 13 (8) | 9 (5) | 14 (9) | 14 (9) | 0.47 |
| Chronic pulmonary disease | 14 (8) | 10 (6) | 13 (8) | 14 (9) | 0.70 |
| Cirrhosis or Hepatic failure | 2(1) | 4 (2) | 7 (4) | 22 (12) | <0.001* |
| Dementia | 4 (2) | 2 (1) | 4 (2) | 4 (2) | 0.73 |
| Diabetes Mellitus | 31 (16) | 33 (16) | 36 (18) | 39 (20) | 0.19 |
| Chronic dialysis | 0 (0) | 0 (0) | 0 (0) | 1(0.5) | 0.17 |
| Ethanol use | 16 (9) | 14 (8) | 26 (15) | 23 (13) | 0.04* |
| Congestive heart failure | 3 (2) | 6 (3) | 6 (4 ) | 5 (3) | 0.47 |
| Hypertension | 50 (29) | 44 (25) | 47 (29) | 54 (34) | 0.32 |
| Immune suppression | 12 (6) | 15 (7) | 17 (9) | 22 (11) | 0.05* |
| Leukemia or Lymphoma | 4 (1) | 11(5) | 7(3.6) | 10 (2) | 0.50 |
| Myocardial Infarction | 10 (6) | 6 (3) | 9 (5) | 5 (3) | 0.39 |
| Peptic ulcer disease | 8 (5) | 10 (6) | 8 (5) | 9 (6) | 0.82 |
| Solid tumor with metastasis | 5 (3) | 2 (1) | 1 (0.5) | 3 (2) | 0.37 |
| Stroke | 4 (2) | 3 (2) | 8 (5) | 10 (6) | 0.03* |
| Peripheral vascular disease | 5 (2) | 3 (2) | 8 (5) | 9 (6) | 0.09* |
| **Baseline Vital Signs** |  |  |  |  |  |
| Temperature (C)  N=798 | 37.8  (37.1-38.3) | 37.6  (37.0-38.4) | 37.7  (37.1-38.3) | 37.2  (36.6-38.0) | <0.001* |
| Mean arterial pressure (mmHg)  N=813 | 74  (66-84) | 75  (68-85) | 75  (68-87) | 74  (67-82) | 0.69 |
| Vasopressor use | 85 (42.5) | 84 (41.4) | 89 (44.3) | 71 (36.0) | 0.30 |
| Central venous pressure (cm H2O) N=753 | 13  (10-16) | 11.5  (9-15) | 11  (8.5-14) | 10  (7-14) | <0.001* |
| Heart rate (bpm)  N=815 | 102  (86.5-118) | 108  (89-119) | 101  (84-116) | 100  (87-116) | 0.06* |
| Fluid intake over prior 24 hours (ml) N=794 | 4266  (2673-6495) | 4515  (2964-7156) | 4462  (2635-6633) | 3433  (2364-5468) | <0.001* |
| Fluid output over prior 24 hours (ml) N=789 | 1925  (1170-3115) | 1740  (1095-2835) | 1891  (1052-3019) | 1669  (1042-2730) | 0.46 |
| Glasgow coma score  N=808 | 8  (3-11) | 8  (3-11) | 8  (3-11) | 8  (4-11) | 0.98 |
| PaO2/FiO2 ratio  N=781 | 113  (80-164) | 124  (84-170) | 113  (79-156) | 120  (83-160) | 0.56 |
| Positive end expiratory pressure (PEEP), N=812 | 10  (5-14) | 10  (7-12) | 8  (5-12) | 8  (5-10) | <0.001* |
| **Severity of illness Scores** |  |  |  |  |  |
| APACHE III  N=784 | 88  (66-1120 | 92  (69-117) | 95  (73-121) | 94  (75-122) | 0.08* |
| Lung Injury Score  N=628 | 3  (2.5-3.3) | 2.75  (2.3-3.3) | 2.75  (2.5-3.3) | 3  (2.3-3.3) | 0.54 |
| **Laboratory values** |  |  |  |  |  |
| Albumin  N=728 | 2.3  (1.9-2.7) | 2  (1.7-2.5) | 2.1  (1.7-2.6) | 2.2  (1.7-2.5) | 0.02* |
| Bicarbonate  N=810 | 22  (19-26) | 23  (19-26) | 22  (19-25) | 22  (18-26) | 0.71 |
| Blood urea nitrogen  N=816 | 18  (13-30) | 18  (11-27) | 17  (12-34) | 18  (11-32) | 0.61 |
| Chloride  N=816 | 107  (104-112) | 108  (104-113) | 108  (103-112) | 107  (102-112) | 0.13 |
| Creatinine  N=816 | 1.1  (0.8-1.6) | 1  (0.7-1.5) | 1  (0.7-1.5) | 0.9  (0.6-1.5) | 0.005* |
| Glucose  N=808 | 127  (104-169) | 120  (103-156) | 125  (100-159) | 120  (98-154) | 0.22 |
| Hemoglobin  N=815 | 10.5  (9.2-11.8) | 10  (9-11.5) | 10.3  (9.2-11.6) | 10  (9-11.1) | 0.04* |
| Platelets  N=772 | 171  (99-247) | 159  (94-260) | 192  (103-272) | 198  (117-271) | 0.11 |
| Potassium  N=816 | 3.9  (3.6-4.3) | 3.9  (3.6-4.2) | 3.9  (3.6-4.4) | 3.9  (3.5-4.5) | 0.49 |
| Sodium  N=816 | 139  (136-142) | 139  (136-142) | 139  (135-142) | 139  (134-142) | 0.19 |
| Total protein  N=713 | 5  (4.3-5.7) | 4.8  (4-4.4) | 5  (4.4-5.7) | 5  (4.4-5.7) | 0.11 |
| White blood count  N=816 | 12350  (7800-16700) | 11800  (7250-17350) | 12380  (7550-19000) | 11600  (7000-16900) | 0.51 |

*p<0.1 criteria for entry into multivariable model

| **Table S2: Characteristics Associated with Change in Adiponectin from Baseline (day 0) to Day 7** | | | | | |
| --- | --- | --- | --- | --- | --- |
| **Characteristic**  Median (IQR) or  N (%) | **Quartile 1**  **(-37281, -2229pg/ml)**  **N=142** | **Quartile 2**  **(-2158, -1320)**  **N=142** | **Quartile 3**  **(1372, 5833)**  **N=142** | **Quartile 4**  **(5924, 88055)**  **N=142** | **p** |
| **Demographics/Anthropometrics** |  |  |  |  |  |
| Baseline Adiponectin (pg/ml)  N=568 | 15498  (9251-26145) | 5682  (2851-8896) | 5172  (2595-9090) | 6947  (4140-13539) | <0.001 |
| Body Mass Index (kg/cm^2^)  N=520 | 26.4 (22.4-31.1) | 29 (25.1-34.1) | 28.3(23.5-33.0) | 26.3(22.9-30.9) | 0.003 |
| Predicted Body weight (kg)  N=567 | 63.8 (55-70.7) | 66.1(54.7-75.3) | 66.1(57-73) | 63.4(54.7-70) | 0.02 |
| Sepsis N(%) | 29 (20.4) | 39 (27.5) | 28 (19.7) | 44 (31) | 0.08 |
| **Comorbid Conditions N(%)** |  |  |  |  |  |
| Cirrhosis | 9 (6.7) | 3 (2.2) | 4 (2.9) | 2 (1.5) | <0.001 |
| Diabetes Mellitus | 28 (20.7) | 30 (22) | 26 (19) | 16 (12) | 0.05 |
| Congestive heart failure | 7 (6.1) | 5 (4.2) | 5 (4.1) | 0 (0) | 0.02 |
| Immune suppression | 9 (6.7) | 4 (2.9) | 14(10.1) | 18 (13.4) | 0.01 |
| Leukemia or Lymphoma | 2(1.4) | 2(1.5) | 6(4.3) | 8(6.0) | 0.04 |
| Stroke | 7 (6.1) | 6 (5.3) | 1 (0.8) | 2 (1.8) | 0.02 |
| **Baseline Vital Signs** |  |  |  |  |  |
| Temperature (C) at baseline  N=554 | 37.6 (36.8-38.3) | 37.6(37-38.1) | 38(37.3-38.5) | 37.3(36.6-38.2) | <0.001 |
| Temperature (C) Change day 7-0  N=510 | -0.4 (-0.9,0.5) | -0.4(-1.1,0.5) | -0.5(-1.15, 0.3) | -0.1(-1.1,1) | 0.09 |
| Systolic blood pressure at baseline (mmHg)  N=567 | 108.5 (97-125) | 112(98-130) | 112(100-127) | 106(94-122) | 0.06 |
| Diastolic blood pressure at baseline (mmHg)  N=567 | 56(50-65) | 58(50-66) | 60(52-70) | 56(50-63) | 0.07 |
| Vasopressor use at baseline N(%) | 43 (30.9) | 60 (43.2) | 55 (38.7) | 72 (51.4) | 0.002 |
| Ability to wean vasopressor day 7-0, N (%) | 32 (24.4) | 42 (32.3) | 43 (32.1) | 54 (40.9) | 0.009 |
| Heart rate at baseline (bpm)  N=567 | 97(86-113) | 100(84-118) | 109(91-118) | 102(89-116) | 0.09 |
| Heart rate change in heart rate day 7-0(bpm)  N=523 | -1 (-20, 7.5) | -9(-24, 5) | -10(-23, 1) | -8(-21, 8) | 0.051 |
| 24hour Fluid intake at baseline (ml) N=552 | 3550 | 4187 | 4751 | 4238 | 0.05 |
| Glasgow coma score change day 7-0  N=557 | 0(0,4.5) | 0.5(-1, 5) | 2(0, 7) | 2(0, 6) | 0.03 |
| PaO_2_/FiO_2_ ratio change day 7-0  N=375 | 37.5 (-17, 100) | 45(1, 93) | 73(21, 119) | 26(-9, 98) | 0.03 |
| Pulmonary artery systolic pressure change day 7-0 (mmHg)  N=95 | 5 (-4, 13) | -2(-11, 5) | -4(-17, 3) | -5(-10, 2.5) | 0.05 |
| **Laboratory values** |  |  |  |  |  |
| Albumin (g/dl)  N=514 | 2.2(1.7-2.7) | 2.2(1.8-2.6) | 2.2(1.8-2.7) | 2.0(1.6-2.3) | <0.001 |
| Bicarbonate change day 0-7 (mmol/L)  N=526 | 4 (0-8) | 5(1-9) | 5(2-10) | 6(2-12) | 0.09 |
| Platelets change day7-0 (K/ul)  N=494 | 26 (-45,135) | 88(-14, 187) | 81(3, 171) | 46(-39, 151) | 0.009 |
| Total protein at baseline (g/dl)  N=502 | 5(4.3-5.8) | 5(4.3-5.6) | 5.1(4.3-5.8) | 4.9(4.2-5.2) | 0.05 |
